# Evaluation of Multi-Resolution Satellite Sensors for Assessing Water Quality and Bottom Depth of Lake Garda

**DOI:** 10.3390/s141224116

**Published:** 2014-12-15

**Authors:** Claudia Giardino, Mariano Bresciani, Ilaria Cazzaniga, Karin Schenk, Patrizia Rieger, Federica Braga, Erica Matta, Vittorio E. Brando

**Affiliations:** 1 National Research Council of Italy, Institute for Electromagnetic Sensing of the Environment, Milano 20133, Italy; E-Mails: bresciani.m@irea.cnr.it (M.B.); cazzaniga.i@irea.cnr.it (I.C.); matta.e@irea.cnr.it (E.M.); brando.v@irea.cnr.it (V.E.B.); 2 Earth Observation and MAPping GmbH & Co.KG, Seefeld 82229, Germany; E-Mails: schenk@eomap.de (K.S.); rieger@eomap.de (P.R.); 3 National Research Council of Italy, Institute of Marine Sciences, Venezia 30122, Italy; E-Mail: federica.braga@ve.ismar.cnr.it; 4 Aquatic Remote Sensing Group, Commonwealth Scientific and Industrial Research Organisation (CSIRO) Oceans and Atmosphere Flagship, Canberra 2601, Australia

**Keywords:** satellite remote sensing, Lake Garda, aquatic optics, remote sensing reflectance, fieldwork activities

## Abstract

In this study we evaluate the capabilities of three satellite sensors for assessing water composition and bottom depth in Lake Garda, Italy. A consistent physics-based processing chain was applied to Moderate Resolution Imaging Spectroradiometer (MODIS), Landsat-8 Operational Land Imager (OLI) and RapidEye. Images gathered on 10 June 2014 were corrected for the atmospheric effects with the 6SV code. The computed remote sensing reflectance (*R_rs_*) from MODIS and OLI were converted into water quality parameters by adopting a spectral inversion procedure based on a bio-optical model calibrated with optical properties of the lake. The same spectral inversion procedure was applied to RapidEye and to OLI data to map bottom depth. In situ measurements of *R_rs_* and of concentrations of water quality parameters collected in five locations were used to evaluate the models. The bottom depth maps from OLI and RapidEye showed similar gradients up to 7 m (*r* = 0.72). The results indicate that: (1) the spatial and radiometric resolutions of OLI enabled mapping water constituents and bottom properties; (2) MODIS was appropriate for assessing water quality in the pelagic areas at a coarser spatial resolution; and (3) RapidEye had the capability to retrieve bottom depth at high spatial resolution. Future work should evaluate the performance of the three sensors in different bio-optical conditions.

## 1. Introduction

Since the 1980s, satellite remote sensing represents an opportunity for synoptic and multi-temporal viewing of water quality of lakes [1–3]. Overall, these applications require sensors which operate in the visible-near infrared wavelengths [4], with high radiometric sensitivity [5] and a spatial/temporal resolution to adequately capture the hydrological and limnological processes in the case study. As a result, most of the work has been more accomplished with the latest generation of ocean colour sensors (*i.e.*, MODIS and MERIS) and with Thematic Mapper (TM), an Earth observing sensor of the Landsat program. The most common methods to retrieve water quality from these sensors have been recently reviewed by Odermatt *et al.* [6]. They provided a comprehensive overview of water constituent retrieval algorithms in for coastal waters and lakes, including empirical approaches and physics-based bio-optical models.

The Moderate Resolution Imaging Spectroradiometer (MODIS) instrument, onboard both Terra and Aqua spacecraft (a NASA-centered international Earth Observing System), provides 12 bit imagery in 36 bands, ranging from 0.4 to 14.4 μm. MODIS is operating since 1999 (2002 for the MODIS onboard Aqua), viewing the entire surface of the Earth every one to two days. Within a viewing swath width of 2330 km, MODIS acquires data at three spatial resolutions (250 m, 500 m and 1 km). In particular, the MODIS dataset at 1 km resolution has been utilised in many studies for assessing the concentrations of water quality parameters in lakes. e.g., Chang *et al.*, Horion *et al.*, and Hu *et al.* [7–9] used MODIS to monitor phytoplankton in Lake Okeechobee (USA), in Lake Tanganyika (East African Rift) and Lake Taihu (PRC) respectively; Kaba *et al.*, and Zhang *et al.* [10,11] assessed suspended particulate matter (SPM) in Lake Tana (Ethiopia) and Lake Taihu (PRC), respectively from MODIS time-series.

The Medium Resolution Imaging Spectrometer (MERIS) instrument provides 12 bit imagery, in 15 bands (from 0.4 to 1.04 μm). MERIS was part of the core instrument payload of the ESA Envisat-1 mission, which has been operating from 2002 to 2012. With a spatial resolution of 300 m, which therefore offered improved possibilities for monitoring of small to medium-sized lakes, the 10-years long record of MERIS imagery have been widely used to assess water quality in many lakes. e.g., Giardino *et al*., Odermatt *et al.*, and Bresciani *et al.* [12–14] in the European peri-Alpine lakes; Matthews [15] in South African inland waters; Ali *et al.*, and Binding *et al.* [16,17] in North America's lakes.

The longest temporal record of satellite imagery suitable for lake studies has been provided by the Landsat program. Landsat data have been acquired routinely for over 40-years: starting with Landsat 4 TM (launched in 1982) and now ongoing with the Landsat-8 Operational Land Imager (OLI), launched in 2013. Although TM shows lower radiometric sensitivity and larger bandwidths with respect to the ocean colour sensors, its spatial resolution of 30 m (combined with a revisiting time of 16 days,) made the sensor attractive for lake studies. Verpoorter *et al.* [18] used Landsat imagery to produce a global inventory of lakes: it contains geographic and morphometric information for ∼117 million lakes with a surface area larger than 0.01 km^2^. Then, water quality of lakes from Landsat has been investigated worldwide; e.g., in Asia [19,20], in Europe [21–23], in North America [24,25], in Africa [26]. Retrospective analyses with Landsat imagery were performed by Dekker *et al.* [27] for benthic cover change detection in a shallow tidal Australian lake and by Lobo *et al.* [28] for mapping the total suspended solids of the Tapajós River (Brazil) from 1973 to 2013. Pahlevan *et al.* [29] observed how the improved design of OLI (with respect to the previous sensors onboard of Landsat) is indeed very promising for inland water studies.

Finally, finer scale studies of aquatic remote sensing have been based on higher-spatial resolution satellite sensors (e.g., QuickBird, Ikonos, WorldWiew-2), although those sensors are known to have inferior signal to noise ratio compared to ocean colour systems [30,31] and are not completely suitable in aquatic remote sensing [32]. Their high spatial resolution (≥5 m) makes those systems very attractive in spatial heterogeneous areas. In particular, many studies [33–37] have shown how those sensors are suitable for mapping bottom properties and depth if *in situ* data for calibrating the algorithms are available.

In this study, we focus on Lake Garda, a large deep Italian lake characterised by clear waters and coastal areas colonised by submerged macrophyte beds. Previous remote sensing studies over Lake Garda mostly used MERIS and Landsat TM imagery to assess water composition in the lake [23,38–40], while airborne imaging spectrometry was used to assess bottom depth and benthic cover [41–43]. In all those studies, the retrieval of water components and bottom properties was achieved with physics-based models, which basically enable the correction of atmospheric effects, the conversion of the water reflectance first into inherent optical properties (IOPs) and then into concentrations of water components such as chl-*a*, SPM and coloured dissolved organic matter (CDOM). The in-water physics-based models were parameterized based on a long term database of ∼150 records collected from 2000 to date [23,43,44]. In case of optically shallow waters the approach also provides information on benthic substrate type and bottom depth.

In this study we evaluated the applicability of currently available satellite sensors to retrieve water composition and bottom depth in Lake Garda. The objectives in this study are: (1) to investigate the suitability of MODIS and OLI to estimate the concentrations of water components in the pelagic areas of the lake; (2) to evaluate for the first time the capability of OLI and RapidEye to retrieve the bottom depth in shallow waters. To all imagery, we applied a consistent physics-based processing chain to convert the radiances measured from satellite sensors into water reflectance, inherent optical properties, concentrations of water constituents and bottom depth according to Bresciani *et al.* [42] and Giardino *et al.* [43]. The models results were evaluated with *in situ* data collected during the satellite overpasses.

## 2. Materials and Methods

### 2.1. Study Area and Fieldwork Activities

Located in the Subalpine ecoregion, Lake Garda is the largest lake in Italy, having an area of 370 km^2^, a water volume of 50 km^3^ and a maximum depth of 346 m. It represents an essential strategic water supply for agriculture, industry, energy, fishing and drinking [45]. Moreover, it is an important resource for recreation and tourism with its attractions of landscape, mild climate and water quality. According to Organisation for Economic Co-operation and Development (OECD) is classified as an oligo-mesotrophic lake [46]: phosphorous concentration in the epilimnium is below or around 10 μg/L, the average concentration of chl-*a* is 3 mg/m^3^, the Secchi disk depths vary between 4–5 m in summer and 15–17 m in late winter [47]. With respect to morphology the lake can be divided in two different areas: the largest sub-basin extended from north to southwest area, characterised by deepest bottoms, and the south-eastern shallower sub-basin. The northern part of the lake is characterized by mountain slopes mainly covered by forests or rural territories, whilst the southern part of the lake is surrounded by morenic and alluvial plains and low hills with a mix of urbanised and rural land use [45].

**Figure 1.**
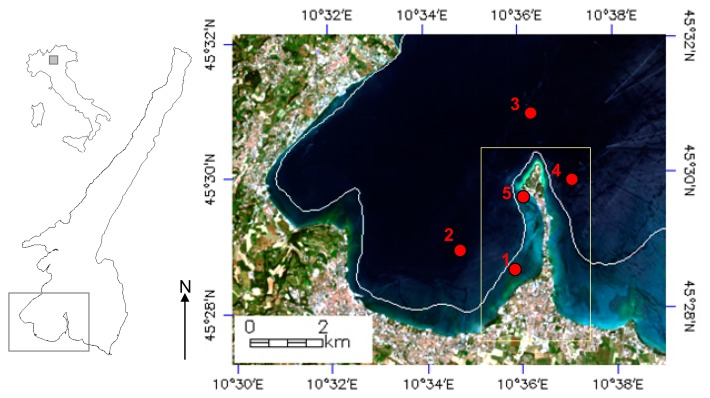
The southern part of Lake Garda (northern Italy) imaged from the Landsat-8 OLI sensor on 10 June 2014 with location of *in situ* stations distributed close the peninsula of Sirmione. The grey-line shows the 7 m bathymetry and the yellow box identifies the study area for bathymetric retrieval.

To perform an assessment of water constituents and bottom retrievals from multi-resolution satellite sensors with the use of match-ups with *in situ* data, a field campaign was conducted on 10 June 2014. A total of 5 investigated stations, distributed in the southern part of Lake Garda (Figure 1), nearby the Sirmione Peninsula extending for about 4 km into the lake. The field campaign focused in the southern part of Lake Garda as it encompasses pelagic waters as well as and a gentle gradient in bottom depth [42]. At each station, Secchi disk (SD) was measured and an integrated water samples between the surface and the SD were collected using a Van Dorn water sampler. Water transparency in the pelagic waters (Stations 2, 3 and 4, Figure 1) was high as the SD depths were equal to 8 m; in station 1 SD was 7 m, which is close to the bottom depth, while in station 5 the bottom was visible and a depth of 3 m was measured.

Water was filtered *in situ* for subsequent laboratory analysis. chl-*a* concentrations extracted with acetone were determined via spectrophotometric method [48]. SPM concentrations were determined gravimetrically [49]. CDOM was determined as the absorption coefficient of CDOM (*a_cdom_*(λ)) at 440 nm according to Kirk [50]. The absorption spectra of phytoplankton *a_ph_*(λ) and non-algal-particle *a_nap_*(λ) were also determined as follows. The absorption spectra of particles *a_p_*(λ) retained onto the GF/F filters were measured using a laboratory spectrophotometer [51]. The filters were then treated with cold acetone (90%) to extract pigments and the absorption spectra of non-algal-particle *a_nap_*(λ) of these bleached filters were measured. The absorption spectrum of phytoplankton *a_ph_*(λ) was derived by subtracting *a_nap_*(λ) from *a_p_*(λ) spectra. In all stations a HydroScat-6 backscattering sensor (HOBILabs, Tucson, AZ, USA) was used to estimate the backscattering coefficient of the particles (*bb_p_*(*λ*)) [52] at 442, 488, 510, 550, 620 and 676 nm. In all stations (expect station 5, cf. Figure 1), remote sensing reflectance (*R_rs_*) values above surface were also measured with a WISP-3 spectroradiometer (Water Insight, Wageningen, The Netherlands) in the optical range of 400–800 nm.

### 2.2. Satellite Image Processing

Synchronous to fieldwork activities satellite images from MODIS, OLI and RapidEye (Table 1) were acquired for 10 June 2014. In order to assess water quality parameters from the radiances measured at satellite levels the physically based approach described by Cracknell *et al.* [52] was adopted. In this approach the concentrations of water constituents (e.g., chl-*a*, SPM and CDOM) are related to the bulk inherent optical properties (IOPs, *i.e.*, absorption and back-scattering coefficients) via the specific inherent optical properties (SIOPs). The IOPs of the water column are then related to the apparent optical properties (e.g., *R_rs_*) and hence to the top-of-atmosphere radiance. These relations are described by the radiative transfer (RT) theory and can be implemented in RT numerical models such as HydroLight-Ecolight [53] and Modtran [54] for in-water (including the bottom in case of shallow waters) and in-atmosphere components, respectively. To determine the water constituents from satellite data, analytical methods based on simplification of RT models can be used [5].

In this study a consistent physics-based processing chain [42,43,55] was applied to MODIS, OLI and RapidEye imagery to enable multi sensor comparisons where the results depend only on the sensor characterisitics (e.g., spatial, spectral, radiometric resolutions, Table 1).

In particular; the vector version of the Second Simulation of the Satellite Signal in the Solar Spectrum (6SV) code [56,57] was adopted to correct the images for the atmospheric effects. The 6SV (version 1) code is a basic RT code; based on the method of successive orders of scatterings approximations and capable of accounting for radiation polarization. An input parameter allows activating atmospheric correction mode. In this case; the ground is considered to be Lambertian; and as the atmospheric conditions are known; the code retrieves the atmospherically corrected reflectance value that will produce the radiance entered as input. The 6SV was executed by an Interactive Data Language (IDL) tool that uses IDL widgets as graphical user interface. Therefore; input data for the 6SV runs were the level 1 satellite radiances achieved from metadata attached to imagery files. The level 1 radiances for OLI data were adjusted using spectral gains suggested by Pahlevan *et al.* [29]. For all images 6SV was run with a mid-latitude summer climate model; an aerosol model suitable for the Lake Garda region and a horizontal visibility of 20 km (±2; depending on the image acquisition time); the latter derived from *in situ* measurements of the aerosol optical thickness. The 6SV-derived atmospherically corrected reflectances were then converted into *R_rs_* (in sr^−1^ units) above water dividing by π.

For each scene the environmental noise-equivalent remote sensing reflectance differences NEΔ*R_rs_*(Δ)_E_, was computed according to Wettle *et al.* [58] to assess the overall sensitivity of the scene signals (depending on sensor, atmosphere and water system) for detecting reflectance changes. Table 1 shows the spectrally-averaged lower level of noise computed on homogenous subsets of pelagic waters. For OLI and MODIS scenes comparable and rather low values of NEΔ*R_rs_*(Δ)_E_ were found. This confirms the findings by Pahlevan *et al.* [29] of high SNR for OLI, whilst the slightly higher value of MODIS is lower than assessed by Hu *et al.* [59] for open ocean waters, as in the Lake Garda image spatial variability in the signal may also be originated by adjacent lands. The RapidEye image has a higher value of NEΔ*R_rs_*(Δ)_E_ which is explained by the lower radiometric sensitivity of the sensor and the higher spatial resolution.

To determine water constituents and bottom depths from satellite-derived *R_rs_*, the spectral inversion procedure implemented in Bio-Optical Model Based tool for Estimating water quality and bottom properties from Remote sensing images (BOMBER) [55] was used. BOMBER is a software package programmed in IDL and uses IDL widgets as graphical user interface. Using semi-analytical models for optically deep and optically shallow waters, BOMBER simultaneously retrieves the optical properties of water column and bottom from remotely sensed imagery [55]. The parameterisation of the bio-optical model implemented in BOMBER was based on a comprehensive dataset of concentrations and SIOPs of Lake Garda waters [42,43,60].

In this study, the discrimination between shallow and deep water was established at 7 m bathymetry. The value is comparable to data gathered from fieldwork activities where an average SD depth of 8 m was measured in bathymetries deeper than 7 m (cf. Figure 1). Moreover, the 7 m depth is also comparable to highest depth at which BOMBER has been used [42,43] to produce reliable estimates of bottom depth in the study area.

The spatial and radiometric resolutions of the sensors where assessed to establish whether the inversion was performed in optically deep and/or in optically shallow waters. As suggested by Dekker *et al.* [21], sensors NEΔ*R_rs_*(Δ)_E_ were used to assess suitability to accurately retrieve water constituents. According to the low NEΔ*R_rs_*(Δ)_E_ values OLI and MODIS *R_rs_* data were spectrally inverted to assess the concentrations of water quality parameters. The RapidEye radiometric sensitivity (NEΔ*R_rs_*(Δ)_E_ = 0.221%) is not suitable for mapping small variations of water constituents that occur in the study area [21]. MODIS was not deemed suitable for the shallow waters analysis as due to the coarse spatial resolution the shallow waters occur mostly in the land-water mixed pixels. RapidEye was considered suitable for mapping shallow waters as the fine spatial resolution allows the bottom depth of southern Lake Garda to be mapped at a high resolution. Based on the resolutions of the three sensors, the retrieval of water quality parameters in optically deep waters was performed only on MODIS and OLI while BOMBER was run for optically shallow waters for OLI and RapidEye to retrieve bottom depths.

## 3. Results and Discussion

### 3.1. Optically Deep Waters

The optically deep waters considered within this study were investigated with OLI and MODIS sensors and *in situ* data gathered from the three more pelagic stations (*i.e.*, 2, 3 and 4, cf. Figure 1).

**Figure 2.**
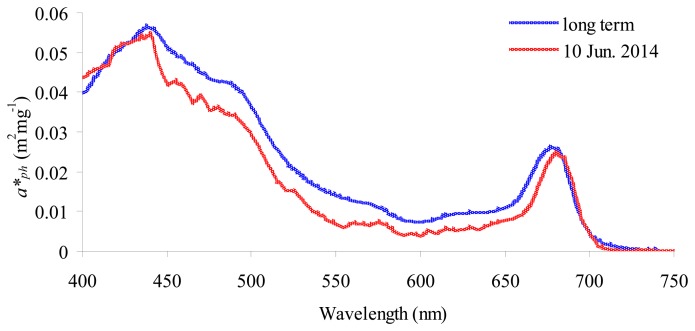
The specific absorption spectra of phytoplankton of Lake Garda.

Widely stable water components conditions were encountered with generally low values of concentrations of water constituents. The measured average values in stations 2, 3 and 4 (cf. Figure 1) were 1.01 mg/m^3^ (±0.32), 0.52 g/m^3^ (±0.13) and 0.03/m (±0.02), for chl-*a*, SPM and CDOM, respectively. The total absorption and backscattering coefficients of particles were also indicating the transparency of water. For instance, at 442 nm, *a_p_* and *bb_p_* were respectively equal to 0.0633 m^−1^ (±0.0200/m) and to 0.013/m (±0.0003). The SIOPs gathered on 10 June 2014 (Table 2 and Figure 2) were consistent with long term SIOPs mean values [42,43]. In particular, the small differences between the spectra of specific absorption of phytoplankton may reflect the low concentrations of chl-*a* compared to long term dataset (ranging between 2.3 and 4.0 mg/m^3^ [47]).

Reliable estimations of water components within such a limited variation range depend on the accuracy and consistency of the parameterization of physics-based processing chain, and it can be assessed with the optical closure between modelled and measured *R_rs_* spectra [5]. Six forward runs of the bio-optical model implemented in BOMBER were performed with all the relevant information gathered from *in situ* observations. In particular, three runs (one for each station) were performed with concentrations of chl-*a*, SPM and CDOM and SIOPs gathered on 10 June 2014 (Table 2 and Figure 2); three other runs (one for each station) were performed with the concentrations of chl-*a*, SPM and CDOM measured on 10 June 2014 and the long term SIOPs (Table 2 [42–44] and Figure 2). The modelled *R_rs_* spectra were compared to the *R_rs_* spectra derived from WISP-3 and atmospherically corrected satellite images respectively. As the change of variation of *R_rs_* spectra between the three stations was very limited, the plot shows the average values only. For MODIS, only the *R_rs_* spectrum corresponding to the most pelagic station (*i.e.*, station 3, cf. Figure 1) was plotted because, for stations 2 and 4, MODIS data were contaminated by the signal coming from the adjacent lands. A feature that, the coarser spatial resolution sensors, already showed in Lake Garda data [40,60].

**Figure 3.**
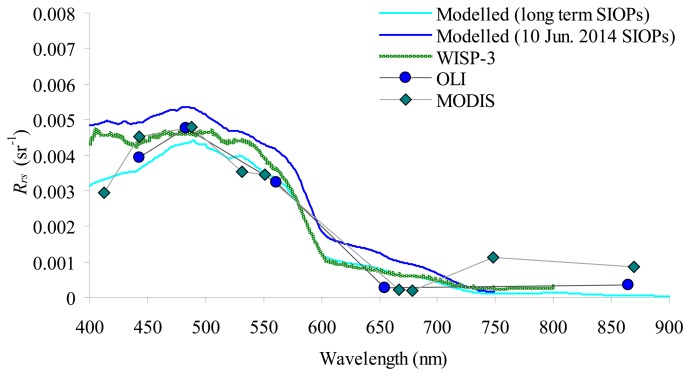
Optical closure on 10 June 2014 in optically deep waters. The *R_rs_* spectra above water are the average value for three stations (*i.e.*, 2, 3 and 4, cf. Figure 1), except for MODIS where the spectra corresponding to station 3 is plotted. The thin continuous lines are the spectra simulated with the bio-optical model implemented in BOMBER from the concentrations of chl-*a*, SPM and CDOM measured on 10 June 2014 together with both the long term SIOPs (cyan line) and the SIOPs measured on 10 June 2014 (blue line).

Figure 3 shows the convergence between modelled and measured *R_rs_* spectra. Overall, the spectra all converge, in particular from 440 to 650 nm where the maximum difference (0.004 sr^−1^) is between MODIS and WISP-3. For the modelled spectra, a small difference in using the long term SIOPs and the SIOPs gathered on 10 June 2014 was observed, reflecting the small differences between the two SIOP sets (Table 2 and Figure 2). The comparison with WISP-3 showed a better closure by using the *R_rs_* spectra modelled with the SIOPs measured on 10 June 2014, the comparison with 6SV-derived OLI and MODIS spectra instead showed a better closure by using the *R_rs_* spectra modelled with the long term SIOPs. The *R_rs_* divergence was slightly higher for MODIS: in the first channel with a drop and at longer wavelengths with an increase of the signal, probably due to adjacency effects which was anyway present in the most pelagic station.

Based on the optical closure analysis we decided to apply BOMBER: (1) with a parameterisation based on the long term SIOPs (the SIOPs measured on 10 June 2014 were used to validate the satellite-inferred estimation); and (2) in case of MODIS, only for the pixel matching station 3 and by excluding in the inversion process the first and the last two bands (*i.e.*, using the 443–675 nm spectral range).

### 3.2. Optically Shallow Waters

The optically shallow waters considered in this study were investigated with OLI and RapidEye sensors and *in situ* data gathered from the two coastal stations (cf. Figure 1): station 1 where SD depth was comparable to the 7-m bathymetry and station 5, where bottom depth was 3 m.

Similarly as for the deeper stations, clear water conditions were encountered. The average concentrations for the two stations for chl-*a*, SPM and CDOM were 0.74 mg/m^3^ (±0.13), 0.74 g/m^3^ (±0.09) and 0.05 m^−1^ (±0.03), respectively. The absorption coefficient of particle *a_p_* at 440 nm and the backscattering coefficient of particles bb_p_ at 442 nm, were respectively equal to 0.0703 m^−1^ (±0.0190 m^−1^) and to 0.018 m^−1^ (±0.0015).

**Figure 4.**
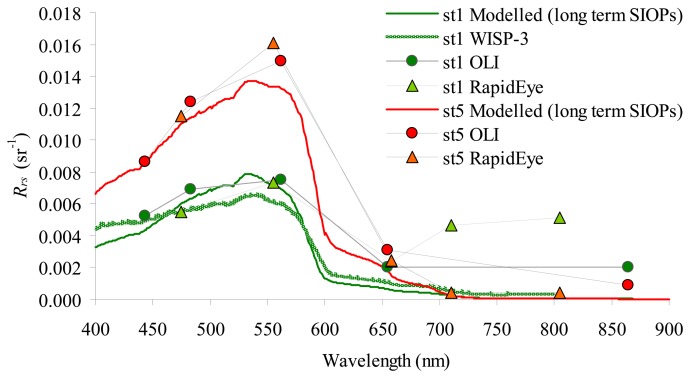
Optical closure on 10 June 2014 in optically shallow waters. The *R_rs_* spectra above water are plotted for two stations at different depth (station 1 at 7 m and station 5 at 3 m). The thin continuous lines are the spectra simulated with the bio-optical model implemented in BOMBER with the concentrations of chl-*a*, SPM and CDOM and bottom depth measured on 10 June 2014 and the long term SIOPs and bottom albedo.

The optical closure in optically shallow waters (Figure 4) was evaluated based on two forward runs of the bio-optical model implemented in BOMBER, with the concentrations of chl-*a*, SPM and CDOM gathered on 10 June 2014 and the long term SIOPs. The runs were also calibrated with bottom depths measured on 10 June 2014 and bottom albedo based on long term data [42]. The two modelled *R_rs_* spectra were compared to the *R_rs_* spectra derived from WISP-3 (for station 1 only) and satellite images, respectively. In both stations the closure was good, both in terms of magnitude and spectral shapes. Only RapidEye in station 1 was diverging because the station is close to optically deep waters and the sensor noise do not allow smaller signals to be detected [43,61].

Based on the optical closure analysis, the BOMBER was considered suitable for inverting the *R_rs_* spectra measured from OLI and Rapid Eye. Following previous studies [62,63] and due to the homogenous conditions of water constituents measured *in situ*, BOMBER was run by keeping constant the concentrations of chl-*a*, SPM and CDOM.

### 3.3. Validation and Mapping

The results produced by applying BOMBER to satellite images, previously corrected for the atmospheric effects with the 6SV code, were compared to the match-ups with in situ data. Table 3 shows the average values (for the three stations in optically deep waters except for MODIS with results for station 3 only), for the concentrations of chl-*a*, SPM and CDOM. Comparable results to *in situ* data were found both from OLI and MODIS, suggesting the capability of the method to assess water quality in clear lake waters.

**Figure 5.**
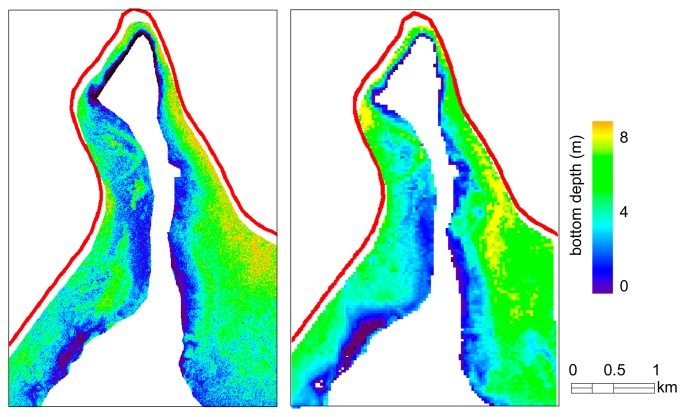
Bottom depth variation from RapidEye (**left**) and OLI (**right**) in the southern part of Lake Garda surrounding the Sirmione peninsula (cf. Figure 1).

Figure 5 shows the bottom depth maps retrieved from OLI and RapidEye, for waters within the 7-m isobath defined by a nautical chart from 1980. The Pearson correlation coefficient *r* of the two maps (with RapidEye image resampled according to the spatial resolution of OLI for a total of 5953 samples) was 0.72. The mapped bottom depths from both OLI and RapidEye reached 8 m, which is acceptable by considering that water level of Lake Garda can change of 1.1 m depending on water use and weather conditions [42]. In correspondence of stations 1 and 5, mapped bottom depths from OLI and RapidEye were comparable to *in situ* observations. In particular, for station 1 (cf. Figure 1), where the SD was 7 m and close to bottom OLI and RapidEye were 6.78 m and 7.10 m, respectively; for station 5, the bottom depth from *in situ*, RapidEye and OLI was 3 m, 3.2 m and 3.8 m, respectively.

## 4. Conclusions

A physics-based approach which allows the concentration of water constituents and bottom depth from satellite images to be retrieved has been applied in southern Lake Garda (northern Italy). The method included the correction for the atmospheric effects with the radiative transfer 6SV code [56,57], the evaluation of the environmental noise-equivalent remote sensing reflectance differences NEΔ *R_rs_*(Δ)_E_ according to [58] and the use of the BOMBER tool [55] for estimating the water related products. The images were acquired on 10 June 2014 from MODIS, OLI and RapidEye sensors. During the satellite overpasses fieldwork activities were conducted to gather data for applying the 6SV code, testing the parameterisation of bio-optical model implemented in BOMBER and examining the imagery-derived products.

Very clear water conditions (SD = 8 m) were observed during the image acquisition date with rather low concentrations of water constituents for the season (chl-*a* = 1.0 mg/m^3^; SPM = 0.52 g/m^3^ and CDOM = 0.03 m^−1^). The NEΔ *R_rs_*(Δ)_E_ analyses suggested that only MODIS and OLI were suitable for assessing such low variation of concentrations. Overall, the results of optical closure showed the good agreement between the 6SV-derived and *in situ* measured *R_rs_* spectra, with highest divergence in the first MODIS band and in the last two RapidEye channels. The results also showed how MODIS was suitable for investigating the most pelagic station only and consequently not adapted to coastal areas and bathymetric investigations. A set of forward runs of the bio-optical model implemented in BOMBER suggested using the long term SIOPs for estimating both water constituents in the optically deep waters and bottom depths in the shallow waters surrounding the peninsula of Sirmione. The BOMBER-derived products showed good match-ups with *in situ* data. OLI and MODIS provided chl-*a*, SPM and CDOM data within the range of *in situ* measurements; the bottom depth maps from OLI and RapidEye were comparable between them (*r* = 0.72) and similar to field observations.

This study indicates that the three sensors used have suitable characteristics to support environmental monitoring in Lake Garda. In particular MODIS was appropriate for assessing water quality constituents in the pelagic areas of Lake Garda. By adopting the calibration proposed by Pahlevan *et al.* [29], OLI was deemed suitable for both optically deep and shallow waters applications as both the spatial and radiometric resolutions enabled a full physics based inversion. Although RapidEye is not specifically designed for aquatic application the study indicated this imagery capability to reproduce lake bottom depth variation.

To confirm the results of this exploratory study, future work should evaluate the performance of the three sensors in different bio-optical conditions. Furthermore, MODIS daily measurements could be used to support of environmental reporting in as demonstrated by Bresciani *et al.* [14] with MERIS data for European perialpine lakes. To achieve this, a quantitative assessment based on an extended match-up analysis using the long term records should be performed both on the method adopted in this study and on MODIS standard product suites.

## Figures and Tables

**Table 1.** Summary of satellite data acquisitions used in this study. The number of bands refers to those used in this study.

**Satellite**	**Data Access**	**UTC**	**Pixel Size (m)**	**Number of Bands**	**NEΔ*R_rs_*(Δ)_E_**
Aqua MODIS	Ocean Colour ^1^	12:50	1000	9	0.018%
Landsat 8 OLI	GLOVIS ^2^	10:04	30	5	0.010%
RapidEye 3 (Choma)	EOLI-SA ^3^	11:13	5	5	0.221%

1 oceancolor.gsfc.nasa.gov;

2 glovis.usgs.gov/;

3 earth.esa.int/EOLi/EOLi.html.

**Table 2.** SIOPs data used in the bio-optical modelling relative to long term mean values data and field measurements gathered on 10 June 2014.

**Coefficient**	**10 June 2014**	**Long Term**
Spectral slope coefficient of the exponential CDOM absorption (nm^−1^) curve	0.025	0.021
Specific absorption of NAP at 440 nm (m^2^/g)	0.031	0.050
Spectral slope coefficient of the exponential NAP absorption (nm^−1^) curve	0.012	0.012
Specific backscattering coefficient of SPM at 555 nm (m^2^/g)	0.0082	0.0071
Backscattering exponent of the power-law SPM curve	0.64	0.76

**Table 3.** Average concentrations (with standard deviation) of water constitutes from *in situ* and satellite images corresponding to three pelagic stations (*i.e.*, stations 2, 3 and 4, cf. Figure 1). For MODIS the estimations are relative to station 3 only.

**Data Source**	**chl-*a* (mg/m^3^)**	**SPM (g/m^3^)**	**CDOM (m^−1^)**
*In situ*	1.01 (±0.32)	0.52 (±0.13)	0.03 (±0.02)
OLI	1.04 (±0.10)	0.69 (±0.08)	0.02 (±0.004)
MODIS	0.83	0.41	0.01
